# State-of-the-Art Update of Pancreatic Cysts

**DOI:** 10.1007/s10620-021-07084-1

**Published:** 2021-08-12

**Authors:** Andrew Canakis, Linda S. Lee

**Affiliations:** 1 Division of Gastroenterology and Hepatology, University of Maryland School of Medicine, Baltimore, MD USA; 2 Division of Gastroenterology Hepatology and Endoscopy, Brigham and Women′s Hospital, Harvard Medical School, 75 Francis St, Boston, MA 02115 USA

**Keywords:** Pancreatic cyst, Pancreatic cancer, Endoscopic ultrasound, Endoscopic ultrasound fine needle aspiration, Intraductal papillary mucinous neoplasm, Serous cystadenoma, Mucinous cystic neoplasm, DNA markers, Biopsy, Ablation, Guideline, Confocal laser endomicroscopy

## Abstract

**Figure:**
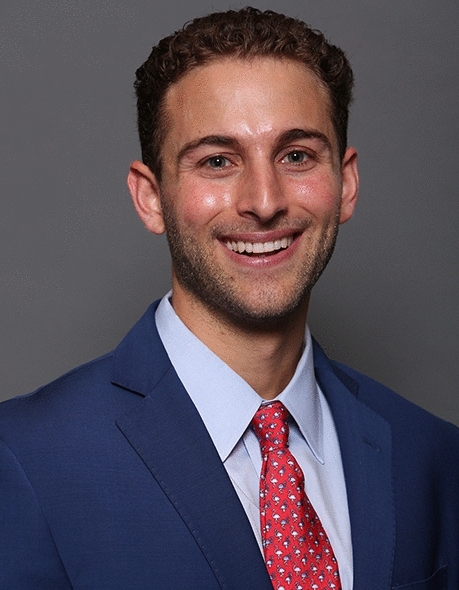
Andrew Canakis

Andrew Canakis

## Introduction

Pancreatic cystic lesions (PCLs) represent a heterogenous group of lesions with varying degrees of malignant potential and a wide clinicopathologic spectrum that mandates careful diagnostic and therapeutic consideration. Numerous guidelines have been developed to assist in the management of these lesions (Table 1) [1–7]. However, the diagnosis and management of PCLs remain problematic due to the variable biological behavior of these lesions, cost of surveillance, morbidity of surgical resection, lack of reliable diagnostics, and need for improved guidelines. Thus, advances in pancreatic cyst diagnostics and therapeutics with endoscopic ablation techniques require further study. Our review aims to shed light on these advances while highlighting the management strategies for PCLs.

**Table 1:** Summary of current pancreatic cyst guidelines

	AGA (2015)	Fukuoka (2017)	ACG (2018)	European (2018)	ACR (2017)
Cyst Type	Incidental cysts	Mucinous cysts	All pancreatic cysts*	All common pancreatic cysts	Incidental cysts
Imaging type	MRI pancreas	Pancreatic protocol CT or MRI pancreas	MRI pancreas	MRI pancreas	Pancreatic protocol CT or contrast MRI
Indications for EUS	At least two high-risk features**	At least one worrisome feature***	At least one worrisome feature***	At least one clinical or radiologic concerning features***	Cyst growth (< 5 mm, 100% growth; 5–15 mm, 50% growth; > 15 mm, 20% growth), peripheral calcification if cyst > 2.5 cm, or ****
Indications to refer for consideration of surgery	Dilated main pancreatic duct + solid component and/or positive cytology	At least one high-risk stigmatal^	At least one concerning symptom or sign, imaging, cytology¥	At least on high-risk stigmata, symptomatic SCA, cystic neuroendocrine > 20 mm, or solid pseudopapillary neoplasm	–

*Except patients with strong family history of pancreatic cancer or genetic mutations predisposing to pancreatic cancer

**AGA high-risk features: cyst diameter ≥ 3 cm, solid component, dilated main pancreatic duct

***Worrisome features: pancreatitis, cyst ≥ 3 cm (≥ 4 cm European), enhancing mural nodule < 5 mm (any size nodule for ACG), thickened cyst wall, main pancreatic duct 5–9 mm, abrupt change in pancreatic duct caliber with distal atrophy, lymphadenopathy, increased serum CA 19–9, cyst growth rate ≥ 5 mm/2 years (≥ 3 mm/year for ACG, ≥ 5 mm/year for European)

**** Nodule, thickened wall, main pancreatic duct ≥ 7 mm, extrahepatic biliary obstruction/ jaundice

^ High-risk stigmata: obstructive jaundice with cyst in head of pancreas, enhancing nodule ≥ 5 mm, main pancreatic duct ≥ 10 mm, suspicious/ positive cytology, MCN (≥ 4 cm European)

¥ Obstructive jaundice due to cyst, acute pancreatitis, elevated serum CA 19–9, nodule of solid component, main pancreatic duct > 5 mm, focal dilation of main pancreatic duct concerning for MD-IPMN or obstructing lesion, ≥ 3 cm IPMN or MCN, HGD or cancer on cytology, solid pseudopapillary neoplasm

With the advent of higher resolution cross-sectional imaging techniques, incidental PCLs have been increasingly discovered. The prevalence in the asymptomatic adult population ranges from 2.4 to 24.3% [6, 8, 9]. Different imaging modalities have reported variable detection rates ranging from 0.2% on ultrasound to over 20% with MRI [10]. De Jong et al. examined 2803 abdominal magnetic resonance imaging (MRI) performed for preventive screening in asymptomatic people in Europe that uncovered 66 (or 2.4%) PCLs, which increased in frequency with age [8]. Individuals younger than 40 years of age, 70 to 79 years of age, and older than 80 years of age had prevalence rates of 0.5%, 11%, and 37%, respectively [8, 9]. Similar findings were seen in an autopsy series by Kimura et al. [11]. While pancreatic cysts are increasingly being discovered, pancreatic cancer-related mortality has not improved. Hence, managing these PCLs has gained considerable attention as clinicians focus on stratifying the malignant potential of these cysts to prevent the mortality associated with progression to pancreatic cancer.

## Common Types of Pancreatic Cysts

**Fig. 1:**
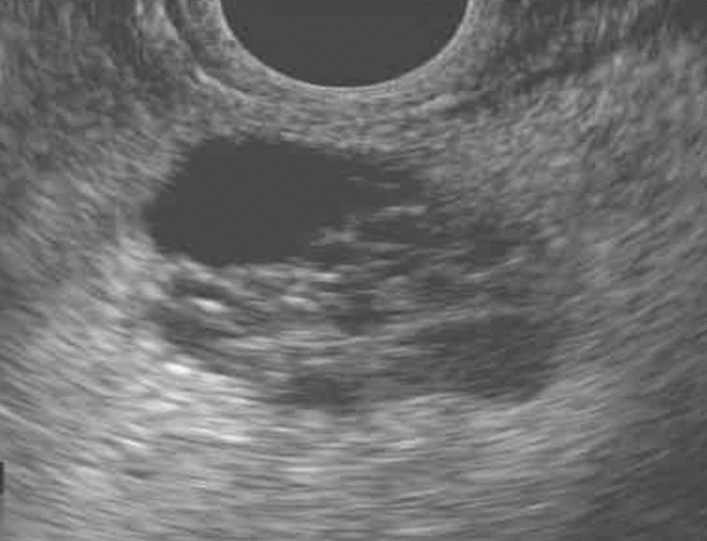
EUS image of lobular, multiseptated serous cystadenoma

These PCLs may be broadly categorized as mucin producing or nonmucinous lesions. Serous cystadenomas (SCA) are one of the most common nonmucinous cysts that are considered benign neoplasms which occur more commonly in women between the fifth and seventh decades of life along the body–tail region of the pancreas (Fig. 1) [12]. Reassuringly, a large retrospective multinational study of 2622 patients found only three serous cystadenocarcinomas in the entire cohort [13]. Unless a patient is symptomatic or the lesion is large, SCAs should be managed conservatively. If confident the lesion is a SCA, no further imaging surveillance is necessary by most guidelines.

**Fig. 2:**
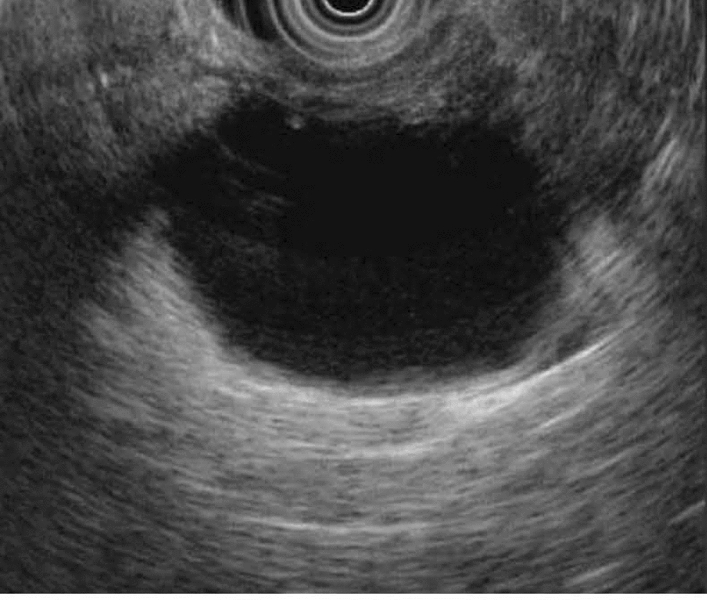
EUS image of unilocular mucinous cystic neoplasm

On other end of the spectrum, mucin producing lesions, such as mucinous cystic neoplasms (MCNs) and intraductal papillary mucinous neoplasms (IPMNs), have malignant potential [6]. MCNs are seen mostly in middle aged women as unilocular lesions in the tail of the pancreas without pancreatic duct communication (Fig. 2), and typically surgical resection is recommended [6, 14]. In a study of 90 surgically resected MCNs, high-grade dysplasia (HGD) and pancreatic cancer were seen in 5.5% and 4.4%, respectively [15]. MCNs carry a 10–39% risk of malignancy [16] although less than 0.4% of cysts less than 3 cm in size without a nodule contain HGD or invasive cancer [17, 18]. Therefore, the European Study Group refined surgical resection criteria for MCN to those with size at least 4 cm, nodule, and/or symptoms [3]. Surveillance is not necessary following surgical resection of MCN unless pathology shows malignancy as these patients have a nearly 25% risk of recurrence (Table 2) [1, 6]. The American College of Gastroenterology (ACG) guideline suggests stopping surveillance after 5 years while the other guidelines do not explicitly recommend stopping surveillance.

**Table 2:** Surveillance Recommendations Following Surgical Resection

Pathology	AGA (2015)	Fukuoka (2017)	ACG (2018)	European (2018)
Invasive cancer or dysplasia	MRI biennially	–	–	–
No HGD or cancer	No MRI surveillance	–	–	–
Invasive cancer in IPMN or MCN	–	Pancreatic cancer surveillance	Pancreatic cancer surveillance (stop after 5 years for MCN)	Pancreatic cancer surveillance
HGD or non-intestinal type IPMN	–	Q6m	Q6m	Q6m × 2 years, then annual MRI
Other IPMN	–	Q6–12 m	Q24m; if IPMN in remnant pancreas, follow non-resected IPMN surveillance	Follow non-resected IPMN surveillance
Non-invasive MCN, serous cystadenoma	–	No MRI surveillance	No MRI surveillance	–

**Fig. 3:**
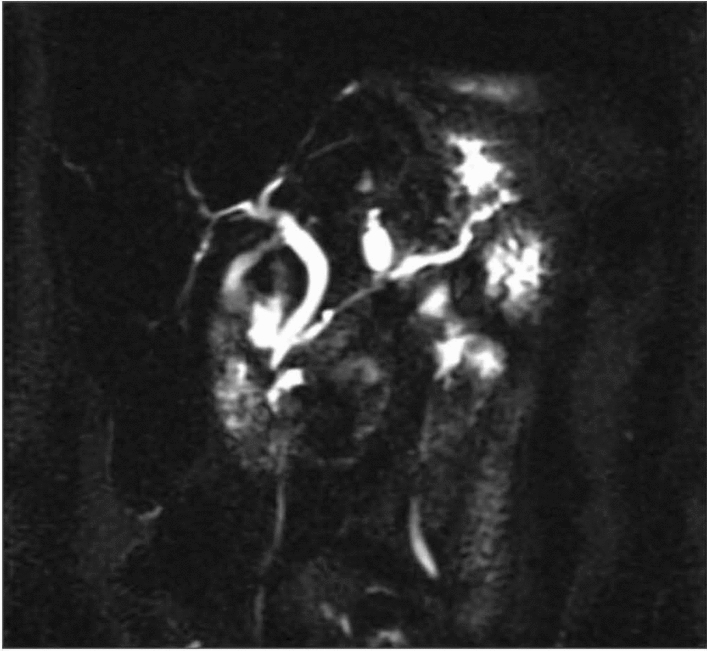
Mixed type IPMN

**Fig. 4:**
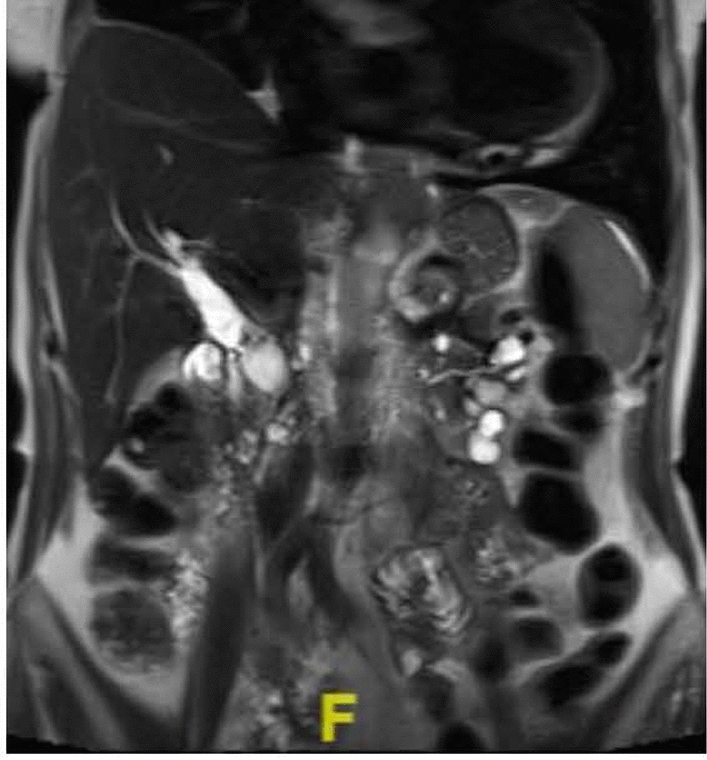
Branch duct IPMN with nondilated main pancreatic duct and cysts scattered throughout pancreas

Intraductal papillary mucinous neoplasms (IPMN) have an equal gender distribution and often occur in the head of the pancreas as solitary or multifocal lesions [16]. IPMNs are radiologically categorized based on their site of involvement within the pancreatic duct: main duct (MD), branch duct (BD), or a combination of both known as mixed (Figs. 3, 4). Malignant potential varies across these different types of IPMNs. MD-IPMNs carry a high risk of malignant transformation of 40 to 90% with 38 to 68% shown to harbor HGD or cancer at the time of resection [6, 10]. Mixed-type IPMNs are thought to have similarly high rates of malignancy (19–68%) although a subtype with only microscopic involvement of the main duct may have favorable rates of malignancy and survival compared with more extensive involvement of the main duct [19]. These high rates of malignancy lead to recommendations for surgical resection of MD-IPMN and mixed-type IPMNs. On the other hand, BD-IPMNs carry a far lower risk (6 to 46%) of malignancy at time of resection with a 22% risk of malignant transformation [1, 12]. Therefore, surgical resection is only recommended in patients with at least one worrisome or high-risk feature. It is important to recognize that not all risk factors carry the same likelihood of malignancy. Five-year survival was 96% for IPMN patients with worrisome features (size > 3 cm, main pancreatic duct 5–9 mm, non-enhancing nodules, cytology with atypical cells, pancreatitis) compared with 60% survival for patients with high-risk features (enhancing nodule, main pancreatic duct ≥ 1 cm, cytology with HGD or cancer, jaundice) [20]. Various guidelines recognize different malignant implications of various features by discussing absolute and relative indications for surgery. The absolute indications for resection by the Fukuoka guideline are main duct dilation ≥ 10 mm, jaundice, enhancing nodule ≥ 5 mm, cytology suspicious or positive for malignancy. The European guideline also includes presence of a solid mass and cytology with HGD or malignancy as absolute indications for surgery [3]. Diabetes may be associated with increased risk of main duct involvement, HGD, and invasive cancer [21].

There remains a lifelong risk from IPMNs [2] as even after resection there is a 0 to 65% risk of recurrence depending on the grade of dysplasia of the resected IPMN [6, 22]. Recurrence includes development of new IPMNs in the remnant pancreas, progression of previously present IPMNs, or occurrence of pancreatic cancer in the remnant pancreas unrelated to an IPMN. Therefore, postoperative surveillance is critical (Table 2).

## Diagnostic Work-Up

### Imaging

Due to variable malignant progression, it is important to characterize these PCLs with high-quality imaging studies to assess for clues of HGD and invasive carcinoma and to determine the type of cyst the patient has in order to guide management. MRI and magnetic resonance cholangiopancreatography (MRCP) are typically preferred over computed tomography (CT) due to lack of radiation and its superior ability to diagnose IPMNs and identify high-risk features. A systematic review of nineteen studies (1,060 subjects) found that CT and MRI differentiated benign and malignant cysts at similarly fair rates (sensitivity for CT 58% to 69% versus 65% to 77% for MRI; specificity for CT 64% to 83% versus 58% to 89% for MRI) [23]. However, MRI was superior for detecting IPMNs (97% vs 81% sensitivity) [23] as well as for detecting pancreatic ductal connections, duct involvement, mural nodules, internal septations, and small cysts [3, 4, 24]. The use of gadolinium-enhanced MRI with MRCP can characterize mucinous features, such as ductal communication, with 91% and 100% sensitivity and specificity, respectively [25]. It is important to highlight several limitations with gadolinium: (1) additional costs, (2) longer times in the scanner which may increase patient anxiety, (3) risk of nephrogenic systemic fibrosis, and 4) the unclear significance of gadolinium deposition in the brain [26]. One MRI surveillance study found that the use of gadolinium did not change clinical management decision in 56 PCLs [27]. Therefore, it may be reasonable to perform surveillance MRI without gadolinium unless concerning features are identified. Secretin-stimulated MRCP may improve anatomical visualization of ductal communication, and Rastegar et al. demonstrated it was identified only by secretin-enhanced studies in 4.7% of patients (*p* = 0.04) [28]. Therefore, secretin-stimulated MRCP may be useful in rare situations to confirm diagnosis of BD-IPMN.

The ability for positron emission tomography (PET) to characterize the metabolic activity and stratify the subtype of IPMNs has proved to be a useful tool in differentiating benign versus malignant lesions with a sensitivity, specificity and accuracy of 80%, 95%, and 87%, respectively [29]. Srinivasan et al. conducted a systemic review and found that PET was superior to the Fukuoka guideline in detecting malignant IPMNs [30]. PET alone is inferior to CT, though the combination of both modalities has been shown to distinguish benign from malignant cysts better than CT imaging alone [23]. A prospective study of 31 cysts (25 benign, 6 malignant) showed that PET/CT was more accurate (94%) than CT (77%) and MRI (87%) imaging for differentiating malignant from benign cysts [31]. However, there is not enough evidence to recommend PET/CT as a go-to imaging technique [6]. All of these imaging studies are subject to misdiagnosis even when reviewer certainty is high [32].

Since MRI, MRCP, and CT have less than 50% accuracy for diagnosing the specific type of cyst in a patient, endoscopic ultrasound (EUS) has emerged as a secondary diagnostic modality to evaluate cysts further [6]. EUS imaging provides visualization of wall thickness, borders, septations, masses, mural nodules, papillary projections, and communication with the main pancreatic duct. However, like CT and MRI, EUS imaging alone is limited in its ability to diagnose lesions with Brugge et al. reporting only 51% diagnostic accuracy for differentiating mucinous from nonmucinous cyts [33]. EUS imaging may be helpful in identifying nodules, which are important prognostic features. Topazian’s group described nodules as hypoechoic with well-defined hyperechoic rim while mucus appeared isoechoic or hyperechoic compared to the adjacent parenchyma with an ill-defined border [34]. Contrast-enhanced harmonic (CH) EUS can effectively differentiate mural nodules from clumps of mucin with low false-negative rates and is more accurate than fundamental B-mode EUS (specificity 75% vs 40% and accuracy 84% vs 64%) [35]. It may also distinguish nodules with HGD or malignancy from low-grade dysplasia (LGD) [35, 36]. A large meta-analysis of 70 studies with 2,297 resected IPMNs showed that presence of nodules identified by CH-EUS had a 62.2% positive predictive value for HGD/ invasive cancer [37]. Nodules taller than 5 mm seem more associated with malignancy although data regarding the implications of nodule size remain limited. CH-EUS may also help in identifying main duct involvement in BD-IPMNs [38]. However, use of CH-EUS remains limited due to several factors: contrast agents remain not FDA approved for use in the pancreas; it is not widely available in the USA; and interobserver agreement remains low. The technique involves intravenous injection of a contrast agent to enhance the microvasculature differences between normal and abnormal tissues. Available agents include perflutren (Definity or Optison) for echocardiograms and sulfur hexafluoride (Lumason) for liver imaging. Following injection, benign lesions enhance to appear hyperechoic, whereas malignant tissues are under-perfused and hypoechoic.

Since EUS is more invasive, it is not recommended for all cysts. Most guidelines suggest the presence of at least one worrisome or high-risk feature before proceeding with EUS (Table 1) [4, 6, 7]. Other reasons to perform EUS include if diagnostic uncertainty remains despite high-quality imaging studies and if it will change management. EUS has ushered in a plethora of opportunities for diagnostic testing of cyst fluid via cytology, pathology, molecular, and chemical analyses (Table 3).

**Table 3:** Common EUS cyst fluid markers

Cyst fluid markers	Sensitivity	Specificity	Comments
Cyst fluid cytology	65%	91%	For malignancy
Cyst fluid cytology	54–63%	88–93%	For mucinous cysts
CEA > 192 ng/mL	75%	84%	For mucinous cysts
CEA < 5 ng/mL	50%	95%	For serous cystadenoma, cystic neuroendocrine tumor, pseudocyst
Glucose < 50 mg/dL	89% to 92%	75% to 86%	For mucinous cysts, pseudocysts
Amylase < 250 U/L	44%	98%	Excludes pseudocysts
KRAS/GNAS mutations	89%	100%	For mucinous cysts

### Cyst Fluid Analysis

#### Cytology

Endoscopic ultrasound-guided fine needle aspiration (EUS-FNA) enables endosonographers to analyze cyst fluid in order to further characterize the PCL in question. The string sign is highly specific for mucinous lesions and can be performed by assessing cyst fluid dripping from the tip of the EUS needle or by placing a drop of fluid between 2 gloved fingers and pulling them apart [39]. It is defined as the fluid extending for at least 1 cm and 1 s.

Lee et al. demonstrated that in a cohort of 603 patients, EUS-FNA is safe and well tolerated with a low complication rate of 2% [40]. Similar findings were reported in a large systematic review of 51 studies, including 10,941 patients, with 0.98% overall complication rate [41]. While rates of infection are very low with a recent noninferiority randomized controlled study confirming similar infection rates regardless of antibiotic prophylaxis, the American Society for Gastrointestinal Endoscopy recommends prophylactic antibiotics when performing EUS-FNA of pancreatic cysts with a fluoroquinolone or comparable antibiotic with similar coverage peri-procedure and then typically continued for 3 days [42, 43].

Cyst fluid cytology detects malignancy with high specificity (91%) but similar to many other cyst fluid markers has low sensitivity (65%) [44]. Two large meta-analyses confirmed similar sensitivity (54% to 63%) and specificity (88% to 93%) for cytology in differentiating mucinous from nonmucinous cystic lesions [45, 46]. The main limitation with cytology is frequent insufficient material to render a diagnosis, which unfortunately occurs in over two-thirds of cases [47]. Performing FNA of the cyst wall after collapsing the cyst may improve diagnostic yield [48]. A recent small study suggested that EUS fine needle biopsy (FNB) provides superior diagnostic yield with acceptable complication rate [49].

#### CEA

Cyst fluid carcinoembryonic antigen (CEA) was the original cyst fluid marker discovered to differentiate mucinous from nonmucinous cysts using 192 ng/ml cutoff with 75% sensitivity and 84% specificity as described by Brugge et al. [33]. In a study of 776 patients, CEA levels in mucinous cysts (4703 ng/mL) were higher than nonmucinous cysts (25.8 ng/mL), and using a CEA cutoff value of 109.9 ng/ml, sensitivity, specificity, and accuracy were 81%, 98%, and 86%, respectively. As the CEA cutoff is raised, the specificity improves at the expense of sensitivity. A systematic review of twelve studies (450 subjects) found levels greater than 800 ng/mL suggested a mucinous cyst with sensitivity and specificity of 48% and 98%, respectively [50]. These different results likely reflect the fact that CEA levels vary depending on which assay is used. Extremely low CEA levels (less than 5 ng/mL) had 50% sensitivity and 95% specificity for SCAs and pseudocyst. While elevated CEA levels can identify mucinous lesions, it is not useful for predicting malignancy [5]. Another limitation of CEA is the minimum volume of fluid required for analysis varies from 0.5 to 1 cc.

#### Glucose

Another biomarker that has been explored to diagnose mucinous lesions is intra-cystic glucose, which has been shown to have similar to superior diagnostic accuracy compared with CEA. A recent meta-analysis reported 91% sensitivity and 75% specificity for glucose compared with 67% sensitivity and 80% specificity for CEA [51]. The most common cutoff of less than 50 mg/dl suggests mucinous cysts with sensitivity ranging from 89 to 92% and specificity 75% to 86% [52–55]. Pseudocysts were also noted to have low glucose levels [53]. Low serum amylase < 250 U/L is highly specific for pseudocysts and may help diagnose pseudocysts when glucose is low. Advantages with glucose measurements include its low cost, rapid and simple measurement even using a glucometer, and small volumes (a few drops of fluid) needed. Whether accuracy for diagnosing mucinous cysts improves with the combination of glucose and CEA remains unclear.

#### DNA-Based Testing

Advances in molecular fluid analysis with DNA-based markers have gained considerable interest as a tool to differentiate mucinous from nonmucinous lesions, characterize mucinous subtypes (IPMN versus MCN), and detect grades of neoplasia. Since the epithelial lining of pancreatic cysts lyses and/or exfoliates DNA into the cyst fluid, assays for various DNA mutations may augment diagnostics. In a retrospective multicenter study, Springer et al. analyzed 130 resected cysts with their molecular sequencing panel, which identified cyst type with 76% to 100% sensitivity and 75% to 100% specificity depending on the specific cyst. This same panel correctly determined cysts needing surgery defined as solid pseudopapillary neoplasm, MCN, or IPMN with HGD or invasive cancer with 75% sensitivity and 92% specificity, which appears more specific than some guidelines [57, 58]. Another study of 225 patients reported their molecular panel testing (KRAS, GNAS, VHL, TP53, PIK3CA, and PTEN) detected advanced neoplasias with a sensitivity and specificity of 100% and 90%, respectively [58].

For identifying mucinous PCLs, KRAS and GNAS mutations have emerged as reliable markers. In a preoperative prospective study of 626 fluid samples using next-generation sequencing, Singhi et al. found that KRAS and GNAS mutations were 89% sensitive and 100% specific for mucinous cysts. Furthermore, presence of KRAS and/or GNAS mutations was 100% sensitive for IPMN while GNAS mutations were 100% specific for IPMN [59–61]. A recent meta-analysis reported that KRAS and GNAS mutations were more accurate for identifying mucinous cysts than CEA alone [62].

While GNAS mutations are not found in MCNs, KRAS mutations appear to increase in prevalence with advancing degrees of dysplasia in MCN with one older surgical pathology study reporting a detection rate of 26% in LGD, 38% in moderate dysplasia, and 89% in HGD or invasive cancer [61]. A recent small study of resected MCNs confirmed higher frequency of KRAS mutations associated with HGD and invasive cancer [63].

On the other end of the spectrum, SCAs do not contain these mutations; instead, VHL mutations and/or deletions occur in 89% to 100% of SCAs [56, 64]. Promisingly, DNA analyses of SMAD4, CDKN2A, TP53, PIK3CA, and PTEN have also been associated with neoplasia [16]. While molecular analysis is costly with uncertain ability to predict cancer risk, it may prove useful when diagnosis remains unclear and a definitive diagnosis would alter management.

### EUS-Guided-Through-the-Needle-Biopsy

Given the diagnostic limitations of cytology and cyst fluid analysis, interest has blossomed for EUS-guided through-the-needle biopsy (TTNB), which offers the possibility of the holy grail of pathology. One such disposable device, a microbiopsy forceps (Moray micro forceps; Steris Healthcare, Mentor, Ohio), is advanced through a 19-gauge needle during EUS-FNA to acquire pinch biopsies of the cyst wall and/or mural nodules. Similar technique may be used with the SpyBite biopsy forceps (Boston Scientific, Marlborough, MA) although there is only a case report using this device [65]. The optimal technique for microbiopsy forceps may be two separate biopsy bites with tenting, which produced 74% diagnostic accuracy [66]. Expert pathologists have substantial to near-perfect agreement when evaluating specimens from microbiopsy forceps [67]. A recent meta-analysis of 9 studies with 454 patients reported diagnostic yield of 69.5% and 88.6% sensitivity for differentiating mucinous from nonmucinous lesions [68]. Technical success is high (86% to 100%) [69–71].

This technique appears to have superior diagnostic yield compared to FNA cytology [72–76]. A meta-analysis of 8 studies with 426 patients noted that TTNB histology was more likely to diagnose the specific type of cyst (72.5%) than FNA cytology (38.1%) as well as mucinous cysts (56.2% v. 29.5%) and SCAs (12.4% v. 1.2%) [77]. Analysis of surgically resected samples in a retrospective study noted 90% concordance with TTNB histology compared with only 20% concordance with FNA cytology [66]. A small comparative study between microbiopsy forceps and conventional analysis (CEA level, cytology, and molecular testing via next-generation sequencing) noted that microbiopsy forceps and standard of care were comparable for identifying mucinous cysts while microbiopsy forceps were superior in diagnosing the specific type of cyst [78].

Despite these findings, there a few limitations to highlight. First, the number of passes needed to acquire adequate samples is not standardized and likely led to varying diagnostic yields and complications. Only one study has examined this issue reaching the conclusion that two separate passes are sufficient [66] while other studies have ranged from performing 1 to 5 passes [71–76, 78, 79]. Also, while diagnostic yield appears significantly improved compared to FNA cytology, it remains modest likely due to sampling issues. The histology samples may not accurately reflect the pathology of the entire cyst in question. A combination of techniques may be necessary to improve diagnostic yield further. Most importantly, adverse events remain a concern. One technical review noted complications ranging from 2 to 23% [80] with meta-analyses reporting a pooled estimate of 7% to 8.6% [68, 77]. A recent prospective study pointed out that although management was changed in 11.9% of cases due to the TTNB results (mostly due to diagnosing SCA), a relatively high rate of complications occurred in 9.9% (10 patients), of which 90% were pancreatitis and 40% severe with 1 death [81]. Therefore, while TTNB is an exciting adjunctive tool in diagnosing pancreatic cystic lesions, the potential for serious adverse events must be weighed against a condition that for most patients will be benign. Further studies are needed to understand when the potential benefits of TTNB in changing management beyond the standard tools of cytology or pathology and cyst fluid analyses outweigh the potential risks.

### EUS-Guided Needle-Based Confocal Laser Endomicroscopy

Needle-based confocal laser endomicroscopy (nCLE) is a novel optical biopsy technique that employs a mini-CLE probe advanced through a 19-gauge needle during EUS-FNA to capture in vivo microscopic images of the cyst’s epithelial lining [82]. Intravenous fluorescein is necessary to enhance blood vessels and surrounding structures. Low-powered laser light is reflected and filtered from the tissue of interest and reconfigured through a computer algorithm to provide magnified, high-resolution images [83].

**Fig. 5:**
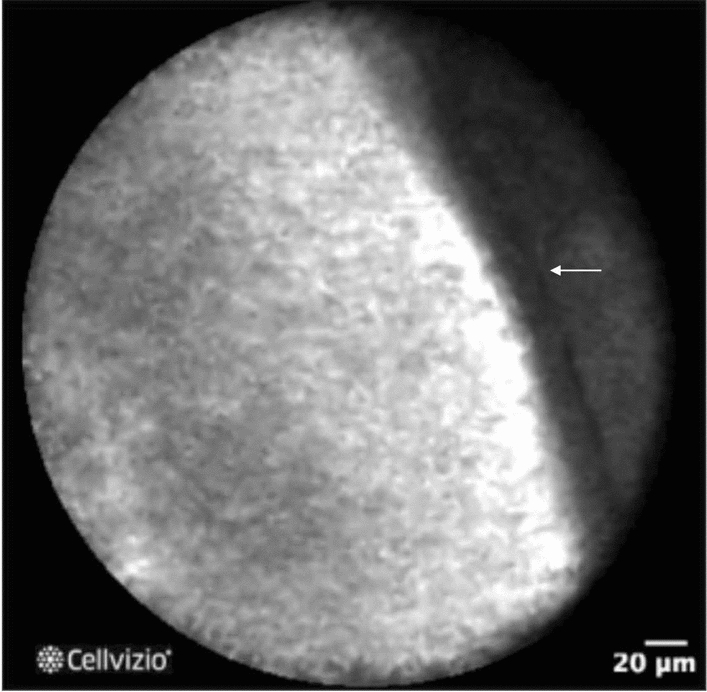
Epithelial band in mucinous cystic neoplasm. Courtesy of Dr. Bertrand Napoléon, CONTACT trials

**Fig. 6:**
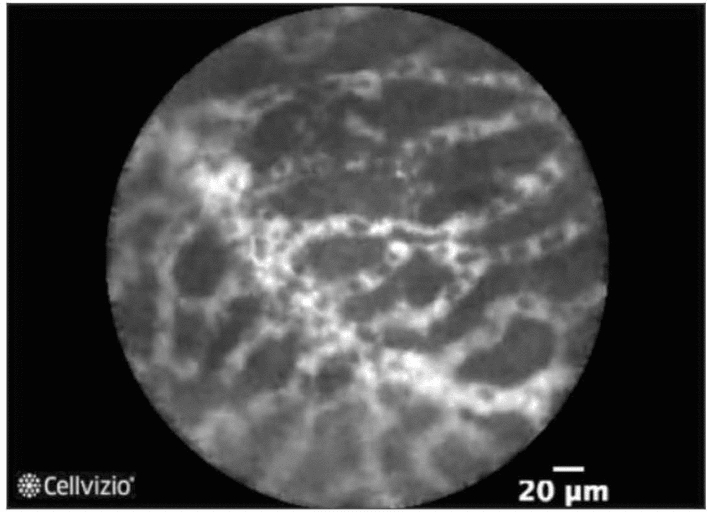
Superficial vascular network in serous cystadenoma. Courtesy of Mauna Kea Technologies

For distinguishing mucinous from nonmucinous cysts, diagnostic accuracy of EUS-nCLE appears superior to CEA (96% v. 64%) or cytology[84, 85] Papillary projections and/or dark rings which are cross-sectional views of the papillae are consistent with IPMNs [86] while thick gray lines are epithelial bands representing MCNs (Fig. 5) [87]. SCAs have a characteristic nCLE finding of a superficial vascular network pattern that microscopically corresponds to dense subepithelial capillaries (Fig. 6) [88]. Studies confirm 97% specificity and 87% sensitivity for diagnosing SCA with this nCLE criteria [85]. One study suggested EUS-nCLE was more accurate than CEA for diagnosing SCA [89]. Cystic neuroendocrine tumors also have very low CEA values, and limited data suggest nCLE may accurately identify these lesions [85]. Findings on EUS-nCLE include dark clumps of cells surrounded by gray areas representing fibrous and vascular areas [87].

A recent study suggests that EUS-nCLE may differentiate LGD IPMNs from IPMNs with HGD or invasive cancer. Papillary width at least 50 $\mu$ m and papillary darkness (cutoff ≤ 90 pixel density) were associated with HGD or invasive cancer, both with 87.5% sensitivity and 100% specificity [90]. Multicenter validation of these criteria will be needed. Artificial intelligence may aid with detection and diagnosis during nCLE. By using convolutional neural network to develop two computer-aided diagnosis algorithms that detected and measured the papillary width and darkness or incorporated various nCLE features, Machicado et al. recently demonstrated improved ability to diagnose HGD and cancer in IPMNs (82.9% and 85.7% accuracy) compared with the Fukuoka (74.3%) or AGA (68.6%) guidelines [91]. Clearly, the impact of artificial intelligence in pancreatic cysts will continue to evolve as the technology does.

There are some varying reports of interobserver agreement for EUS-nCLE. Apart from an outlier study that reported poor to fair global interobserver agreement with mean diagnostic accuracy of only 46% (ranging from 20 to 67%)[92], other studies have suggested moderate to substantial agreement for identifying mucinous cysts and substantial to almost perfect agreement for SCAs [87, 92–94]. Some critiques of the Karia et al. study[92] include short video clips, few patients with surgical pathology confirmed diagnoses, and assessment of findings that are not the validated criteria for various PCLs [85].

Adverse events from EUS-nCLE range from 0 to 9% with a pooled 2.9% risk of acute pancreatitis with 1 severe case [85, 86, 95]. To minimize post-procedure pancreatitis, these techniques are recommended although confirmatory studies are lacking to prove their efficacy in reducing risk: (1) Keep duration of nCLE as short as possible and minimize catheter manipulation. As soon as diagnostic nCLE criteria are visualized, the probe should be removed; (2) Perform EUS-nCLE for no longer than 6 min; (3) Do not brush the tip of nCLE catheter across the epithelial surface to interrogate a different area. Instead, pull the instrument back, and then, gently place the catheter tip on the new area as perpendicular as possible to the epithelial surface [85]. Despite using this technique, a limitation of nCLE is that the entire cyst wall cannot be visualized (at best, approximately 30% to 50% can be scanned) [96]. However, the same limitation occurs with performing EUS-TTNB. A single-center retrospective study compared standard of care (clinical, morphologic, cyst fluid cytology, and CEA analysis) to TTNB and nCLE, noting that diagnostic yield for each modality (75% and 84.1%, respectively) was greater than standard of care (34.1%) [97]. Combining either or both techniques with standard of care led to similar diagnostic yields. An issue with this study is that other cyst fluid markers including glucose, amylase, and DNA markers were not incorporated in the standard of care arm.

The place of EUS-nCLE in the algorithm for evaluating PCLs remains to be determined. While studies have suggested its superior accuracy compared with CEA or cytology, no studies have compared all the available cyst fluid markers of CEA, glucose, DNA markers, and FNB to nCLE. Different approaches may be taken for incorporating nCLE into practice: EUS-nCLE may be performed in all patients with indeterminate cysts undergoing EUS or selectively following an initial nondiagnostic EUS-FNA. Wide dissemination of this technology remains limited due to need for training and costs. Endosongraphers must be competent in pancreatic EUS and EUS-FNA before training in nCLE. The recent expert consensus statement recommends a minimum of 10 supervised procedures to achieve competency and ongoing annual performance of at least 10 EUS-nCLE to maintain competence [85]. Adverse events like pancreatitis have been shown to decrease with increased operator experience [94]. A health economic evaluation of nCLE in France reported that EUS-FNA plus nCLE could reduce 23% of surgeries which would translate into a 13% reduction in clinical costs in the public sector [98]. However, further studies are needed to understand both the optimal clinical niche and financial implications of nCLE.

## Approach to Pancreatic Cyst

**Fig. 7:**
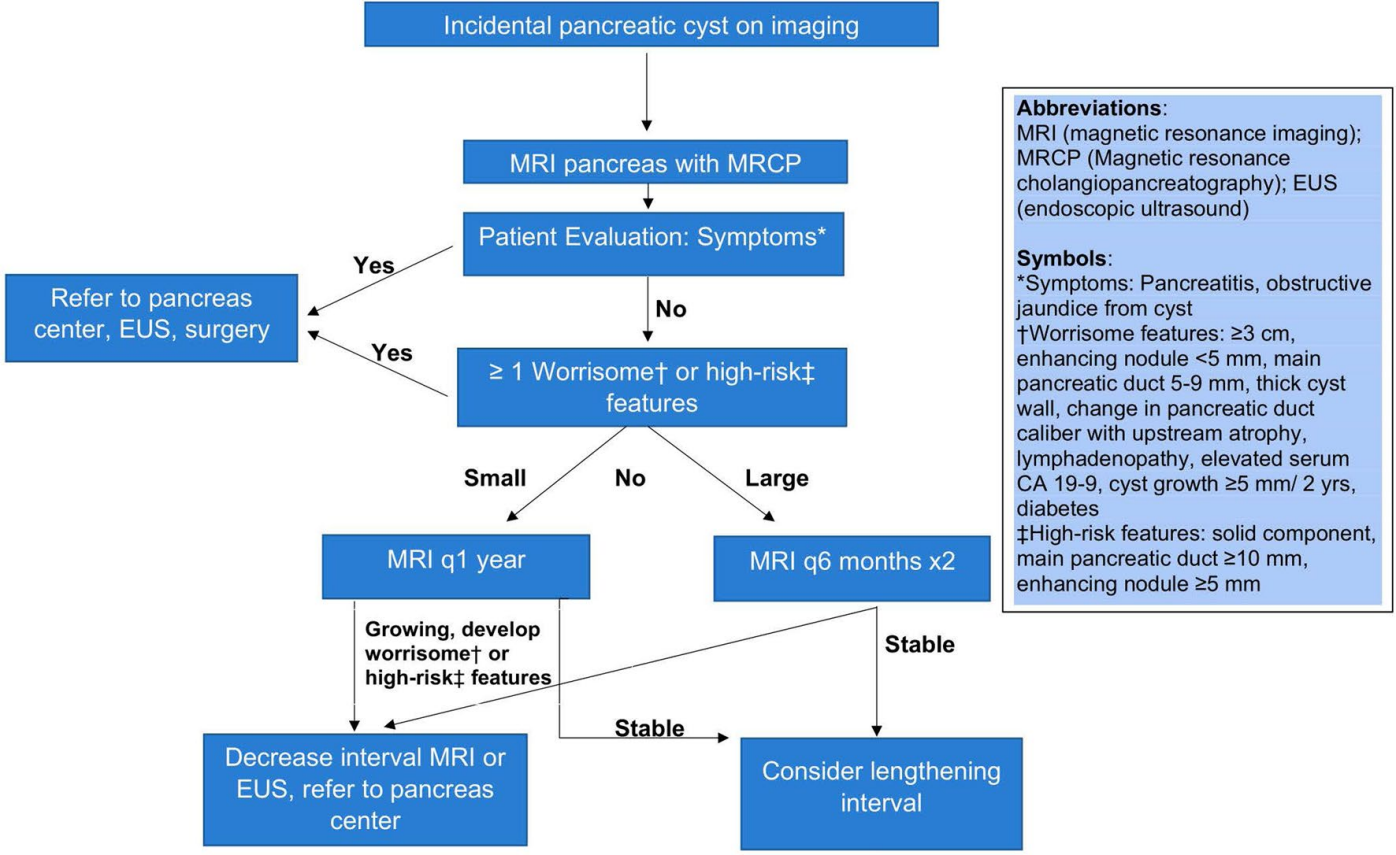
Incidental Pancreatic Cyst Management Algorithm

Despite these advances in diagnostic techniques, managing PCLs is a constantly evolving topic that revolves around stratifying the malignant potential of the cyst. The authors’ suggested algorithm for approaching incidental pancreatic cysts provides a framework for approaching these lesions (Fig. 7). However, before proceeding with this management paradigm, it is important to determine if the patient even requires further evaluation—Are they medically fit for surgery or is the cyst an asymptomatic non-neoplastic (pseudocyst) or benign lesion (SCA) that requires no follow up? Patients with IPMNs and high Charlson Comorbidity Index (≥ 7) had significantly shorter survival (43 months) compared to 180 months for patients with lower scores and had an 11-fold higher risk of dying from non-IPMN-related causes within 3 years [99]. A similar study using the Charlson Comorbidity Index noted that high-risk patients with low-risk cysts were more likely to die from non-pancreatic cancer [100]. Another scoring system (Adult Comorbidity Evaluation 27) was used in a study of 793 patients with IPMNs and reached similar conclusions that patients with higher scores were more likely to die from non-IPMN-related causes [101]. Thus, these scoring systems may assist clinical decision-making when significant comorbidities are present.

The AGA guideline with its controversial recommendation to stop surveillance after 5 years of stability engendered a flurry of long-term studies of pancreatic cysts. A long-term retrospective study found a very low rate of malignant progression (0.9%) for 108 BD-IPMN ≤ 1.5 cm that were followed for more than 5 years [102]. However, another study following BD-IPMNs without worrisome or high-risk features for a minimum of 5 years showed that worrisome or high-risk features developed in 18% of BD-IPMNs well beyond 5 years of observation [103]. Similar findings were observed in a cohort of 1,036 BD-IPMNs without worrisome features in which 4% and 1% of cysts developed worrisome features and pancreatic cancer, respectively, after a median 62 months [104]. In light of these studies, surveillance cannot end in all patients after 5 years, and whether there is a subset of patients in whom surveillance may be safely stopped requires further investigation.

While no study has yet proved reduction in mortality with surveillance, the risk of malignancy cannot be ignored, and it is critical that shared decision-making with the patient is pursued when offering surveillance. Issues to consider include (1) the psychological distress associated with annual surveillance and (2) the significant financial burden to the health-care system. One study estimated that if all patients with cysts (aged 40–79) underwent MRI surveillance, the median cost would be $9.3 billion per year [105]. These calculations did not factor in the cost of surgery or EUS. There is also the concern that patients can be lost to follow up with recent studies finding that 47% to 71.5% of patients received follow-up imaging for incidental cysts [106, 107]. In a large urban safety net hospital, access to gastroenterology care was associated with surveillance completion [106]. More specifically, referral to a multidisciplinary pancreas group is recommended for cysts with any concerning features and when considering surgical resection as evaluation by such a center changed management in 30% of patients away from surgery and toward surveillance [108]. Pancreatic resection should be performed by experienced surgeons at these high-volume centers due to significantly lower post-surgical mortality rates (1–5%) compared with low-volume centers (11–15%) [4, 6].

Patient education about their cysts is important as a survey found that 96% of patients were unaware of their specific cyst type and 58% did not realize some cysts had malignant potential [109]. Physicians need to help patients understand the risks and benefits of pursuing imaging surveillance.

## EUS-Guided Ablation Techniques

The need for minimally invasive treatment of PCLs has led to the development of EUS-guided cyst ablation, designed to destroy the neoplastic epithelial lining of the cyst in patients who decline or are not operative candidates. Cyst ablation can be achieved by the injection of ethanol, antitumor agents (paclitaxel), or radiofrequency ablation (RFA). Although in its infancy, EUS-guided ablation has been shown to resolve cysts in 33% to 79% of cases while also improving quality of life by avoiding surgery [10]. Moreover, propensity score matching analysis found that compared to PCLs undergoing surveillance initially (*n* = 428), the EUS alcohol ablation group (*n* = 118) had significantly lower surgical resection rates (4.8% vs 26.2%) with similar survival over mean follow-up of over 6 years [110].

Patients eligible for cyst ablation by injection of ethanol or antitumor agents include those with a mucinous cyst larger than 3 cm or over 2 cm and growing while malignancy and short life expectancies are contraindications [10, 111–113]. Ideal cysts for EUS-guided ablation have the following features: unilocular or oligolocular (less than 7 locules) to ensure each locule is injected appropriately [10]; cyst size 2–6 cm; and no open communication with the main pancreatic duct.

Response to ablation is defined by volume reduction in the cyst, which is susceptible to variations in cyst measurement even when using the same modality pre- and post-procedure. Complete response is defined as at least 95% reduction in volume, partial response as 75% to 95%, and non-response as 0% to 74% [113].

### EUS-Guided Alcohol Ablation

As a widely available, inexpensive, low-viscosity agent with quick onset of action, ethanol is an effective ablative agent to treat cystic lesions throughout the body [112]. Shortly after injection, it induces cell membrane lysis, protein denaturation, and vascular occlusion [10]. Studies using ethanol ablation to treat PCLs have reported complete resolution rates ranging from 9 to 85% [114–119]. This wide range is likely related to different study designs, techniques, types of cysts treated, and levels of ethanol concentration used, which varied from 80 to 100%. In addition to the wide variability in outcome, adverse events range from 3.3% to 33.2% with pancreatitis occurring in 3.3% to 9.8% [118–120]. These studies raise concern over the long-term efficacy and safety of ethanol ablation. *EUS-Guided Alcohol and Paclitaxel Ablation*:

As a chemotherapeutic agent, paclitaxel is highly viscous and hydrophobic and can theoretically remain in the cyst for a long time to exert its apoptotic effects along the epithelial lining. A prospective study of 164 patients who underwent cyst ablation with alcohol and paclitaxel reported complete resolution, partial resolution, and persistent cyst in 72%, 20%, and 8%, respectively [121]. The 114 patients with complete resolution were followed for 6 years with only 1.7% cyst recurrence [121]. Cyst diameter < 35 mm [122] and absence of septa [121] were predictors of complete resolution. Needle punctures through septa can improve ablative outcomes while minimizing adverse events [123]. Interestingly, DeWitt and colleagues found that baseline DNA mutations can be eliminated in 72% of post-ablation cyst fluid [124].

This combination therapy has replaced ethanol as the preferred approach for cyst ablation both due to increased efficacy and decreased complications [121–128]. Pooled adverse events are lower for paclitaxel-based regiments (15%) compared to ethanol lavage (21.7%) [129]. Pancreatitis has been attributed to the extravasation of ethanol. Therefore, the question that naturally follows is whether alcohol is necessary for cyst ablation and whether adverse events can be reduced without alcohol. In a randomized trial comparing paclitaxel and gemcitabine with and without 80% ethanol, all adverse events occurred in the alcohol group (22% minor and 6% serious events) [128]. Complete ablation occurred at similar rates in both groups (67% in alcohol-free versus 61% in alcohol control group). A larger NIH-funded study is ongoing to validate these findings [130]. Future studies may lead to the development of new delivery agents, improved techniques, and standardized definitions of clinical success that will hopefully broaden the application of EUS-guided cyst ablation.

### Radiofrequency Ablation

Radiofrequency ablation (RFA) is a safe, effective, and well-established technique used to treated cystic lesions (such as in the thyroid and kidney) [131, 132]. Currently only a 19-gauge EUS-guided RFA needle is available to induce direct cell death via coagulative necrosis and hyperthermic energy [133, 134]. This necrotic cascade releases intracellular antigens that activates a systemic immune response (hours to days following the procedure) [135]. Only a few studies have investigated the role of EUS-RFA in treating PCLs [133, 134].

In 2015, Pai and colleagues conducted a preliminary study of EUS-RFA in which all 6 PCLs were treated successfully with 2 cysts having complete resolution and 3 demonstrating a 48.4% size reduction 3–6 months later [134]. There were no major adverse events and only two cases of mild self-limited abdominal pain. A larger prospective study by Barthet et al. treated and followed 16 BD-IPMNs and 1 MCN over a 1-year period [133]. The overall rate of adverse events was 10%, but a modified protocol decreased the complication rate to 3.5%. Pancreatitis complicated by infection and jejunal perforation occurred in the first two study participants. Subsequent protocol modifications of using rectal diclofenac, antibiotics, and cyst fluid aspiration prior to RFA led to only one subsequent complication (pancreatic duct stenosis). Among the 17 PCLs, complete resolution at 6 and 12 months occurred in 47% and 64.7%, respectively. This delayed response may be secondary to the immunostimulatory effects of RFA. In one small study of SCAs, EUS-RFA led to 61.5% radiologic response with median volume of cysts decreasing from 37.82 mL to 10.95 mL [136]. Clearly, larger- and longer-term studies are needed to further assess the efficacy and safety of EUS-RFA in pancreatic cysts.

## Conclusion

Pancreatic cysts remain a diagnostic and management challenge to clinicians and require shared decision-making with patients and occasionally a multidisciplinary approach. While the majority of cysts may be followed with MRI likely without gadolinium, careful appreciation for when to stop surveillance in higher-risk patients, when to refer for EUS in the presence of worrisome or high-risk features or indeterminate cysts, and when to consider surgical resection is essential. The exciting potential of newer diagnostic tools including through the needle biopsy and needle-based confocal laser endomicroscopy as well as novel therapeutics with EUS-guided ablation techniques requires further study.

## Key Findings and Unmet Needs

1. Pancreatic cystic lesions are commonly discovered due to advances in cross-sectional imaging and increased use of abdominal imaging and often represent a diagnostic dilemma for clinicians.
2. Risk stratifying the malignant potential of an incidental pancreatic cyst to determine the optimal next steps requires an individualized approach informed by the guidelines, with some patients benefiting from referral to multidisciplinary pancreas centers.
3. Advances in molecular fluid analysis with DNA-based markers have gained considerable attention as a tool to diagnose mucinous lesions with further work necessary to determine the optimal markers for predicting grades of neoplasia.
4. On the other hand, measuring the humble cyst fluid glucose level also appears to improve identification of mucinous cysts.
5. Newer endoscopic ultrasound-based diagnostic tools, needle-based confocal laser endomicroscopy and microbiopsy forceps, are exciting, but require further study regarding their safety profile and role in the diagnostic algorithm.
6. Endoscopic ultrasound ablation techniques have led to varying degrees of cyst resolution, but further study is necessary to determine the optimal technique and long-term durability.

## Abbreviations

PCLs: Pancreatic cystic lesions
MRI: Magnetic resonance imaging
SCA: Serous cystadenoma
MCN: Mucinous cystic neoplasm
HGD: High-grade dysplasia
IPMN: Intraductal papillary mucinous neoplasm
MD: Main duct
BD: Branch duct
MRCP: Magnetic resonance cholangiopancreatography
CT: Computed tomography
PET: Positron emission tomography
EUS: Endoscopic ultrasound
CH: Contrast-enhanced harmonic
EUS-FNA: Endoscopic ultrasound-guided fine needle aspiration
LGD: Low-grade dysplasia
CEA: Carcinoembryonic antigen
AGA: American Gastroenterological Association
EUS–TTNFB: EUS-guided through-the-needle forceps biopsy
nCLE: Needle confocal laser endomicroscopy
QoL: Quality of life
